# In vivo imaging of brown adipose tissue vasculature reactivity during adrenergic stimulation of non-shivering thermogenesis in mice

**DOI:** 10.1038/s41598-022-25819-6

**Published:** 2022-12-10

**Authors:** John C. Garside, Eric W. Livingston, Jonathan E. Frank, Hong Yuan, Rosa T. Branca

**Affiliations:** 1 Department of Physics and Astronomy, University of North Carolina at Chapel Hill, Chapel Hill, NC USA; 2 Biomedical Research Imaging Center, University of North Carolina at Chapel Hill, Chapel Hill, NC USA; 3 Department of Radiology, University of North Carolina at Chapel Hill, Chapel Hill, NC USA

**Keywords:** Biophysics, Physics, Translational research

## Abstract

Brown adipose tissue (BAT) is a fat tissue specialized in heat production (non-shivering thermogenesis) and used by mammals to defend core body temperature when exposed to cold. Several studies have shown that during non-shivering thermogenesis the increase in BAT oxygen demand is met by a local and specific increase in tissue’s blood flow. While the vasculature of BAT has been extensively studied postmortem in rodents using histology, optical and CT imaging techniques, vasculature changes during stimulation of non-shivering thermogenesis have never been directly detected in vivo. Here, by using computed tomography (CT) angiography with gold nanoparticles we investigate, non-invasively, changes in BAT vasculature during adrenergic stimulation of non-shivering thermogenesis by norepinephrine, a vasoconstrictor known to mediate brown fat heat production, and by CL 316,243*,* a specific β_3_-adrenergic agonist also known to elicit BAT thermogenesis in rodents. We found that while CL 316,243 causes local vasodilation in BAT, with little impact on the rest of the vasculature throughout the body, norepinephrine leads to local vasodilation in addition to peripheral vasoconstriction. As a result, a significantly greater relative increase in BAT perfusion is observed following the injection of NE compared to CL. This study demonstrates the use of in vivo CT angiography as an effective tool in assessing vascular reactivity in BAT both qualitatively and quantitatively in preclinical studies.

## Introduction

Since a series of studies confirmed the presence of active brown adipose tissue (BAT) in adult humans^1–4^, interest in understanding BAT’s distribution, activity, and metabolic impact has been significantly revitalized^5,6^. The primary function of BAT is non-shivering thermogenesis (NST), a norepinephrine-stimulated process wherein UCP1, a protein present at high concentration in the mitochondria of BAT, dissipates the proton gradient created by the electron transport chain to generate heat rather than ATP^7–10^. BAT’s energy expenditure during NST, which in humans has been estimated to account for up to 15% of total energy expenditure, and glucose uptake capacity makes this tissue an attractive target for the treatment of obesity and diabetes^11,12^.

The vasculature of BAT is known to play an essential role for NST. Compared to neighboring white adipose tissue (WAT), active BAT is more innervated and vascularized. It has been shown that by stimulating angiogenesis and the conversion of WAT to brown-like adipocytes, weight gain in obese mice can be inhibited^13^. Conversely, the development of obesity in mice by overnutrition caused decreased vascular density in BAT, which then caused whitening of the fat^14^.

Intravascular infusion of latex and polyvinyl resins has been used to study the vasculature supplying and draining the interscapular BAT (iBAT), the prominent BAT depot found in rodents^15^. It was found that blood vessels such as Sulzer’s vein and the thoracodorsal artery were among the more prominent vascular structures in the area^16^. BAT vasculature has also been examined in mice postmortem, after infusion of NE or saline, using light microscopy following latex casting of the BAT vasculature tree^17^. It was shown that BAT of mice injected with NE was significantly more perfused than BAT of mice infused with saline. More recently, micro-CT scanning with a gold nanoparticle-based blood pool contrast agent, Aurovist, has been used also postmortem in mice to produce high resolution images of the vasculature around iBAT and quantify vascular diameters^18^. Yet, in vivo imaging of dynamic vascular changes in response to BAT stimulation has never been reported.

When studying BAT, it is important to understand not only the anatomy of the vasculature around BAT, but also how the vasculature, and consequently perfusion, changes in response to the stimulation of BAT. Indeed, NST is known to elicit significant increase in BAT perfusion, both to respond to the increase in oxygen demand of this tissue, as well as to efficiency redistribute the heat produced^19,20^. As a result, reduced vasculature tone may be a limiting factor in BAT’s ability to undergo NST^21^. Blood flow through a vessel can be modeled by Poiseuille’s Law, $Q= \frac{\pi P{r}^{4}}{8\eta l}$ , where $Q$ is volume flow rate, $P$ is the pressure difference across the vessel, $r$ is the radius of the vessel, $\eta$ is the viscosity of blood, and $l$ is the length of the blood vessel^19,22^. NST thermogenesis is known to increase heart rate^23^, which inherently increases the rate of blood flow. Poiseuille’s law also demonstrates that an increase in pulse pressure, or the difference between systolic and diastolic blood pressure, increases blood flow through a vessel.

An important mechanism by which BAT increases local perfusion is through vasodilation. In response to an increased need for oxygen or nutrients, tissues release endogenous vasodilators, which lead to a decrease in vascular resistance and increase in perfusion. Vasodilation and vasoconstriction (vascular tone) is generally regulated by adrenergic receptors, membrane proteins that can be divided into α and β classifications^24^. Adrenergic agonists cause a wide range of physiological effects, but generally, binding to α adrenergic receptors results in vasoconstriction while binding to β adrenergic receptors results in vasodilation^24,25^. All three subtypes of β adrenergic receptors are found in the brown adipocytes of mice, but BAT is unique in that it is the primary location of highly concentrated β_3_ receptors^26^, at least in mice. Stimulation of BAT with norepinephrine (NE), a broad α and β adrenergic receptor agonist, activates NST^7,27^ and increases blood flow to the tissue^17,28^. Similarly, CL-316,243 (CL), a highly selective β_3_ agonist, is another molecule known to activate BAT that can induce browning in adipocytes over time^29,30^. It has been shown that brown adipocytes that are activated by either NE or CL may produce nitric oxide (NO), which triggers local vasodilation by relaxing vascular smooth muscle^31,32^. Additionally, stimulation of β_3_ receptors in BAT releases adiponectin^33^, resulting in the production of NO in nearby vascular endothelial cells and consequently vasodilation^34^.

Here, by using whole-body high-resolution Computed Tomography Angiography (CTA), we report the first direct detection of vasculature changes, both local in BAT and in whole body, that occur during adrenergic stimulation of BAT thermogenesis in mice in vivo. CT is a tomographic imaging technique that enables the acquisition of high-resolution images in just few seconds or minutes, depending on the needed resolution, signal to noise ratio, and field of view. In CT images, contrast is originated by tissue radiodensity, i.e. by the ability of tissues to absorb or scatter radiation. Tissue radiodensity is measured in Hounsfield Units (HU), a quantitative scale used to differentiate tissues. In absence of exogenous contrast, soft tissue radiodensity is generally determined by the relative concentration of water and fat in the tissue. For this reason, in non-contrast CT images, BAT can be easily differentiated from WAT as its radiodensity is generally higher than that of WAT but lower than that of muscle. This is true at least in mice housed a normal room temperature, in which chronic mild cold exposure^35^ leads to a relatively high hydrated BAT (< 60% fat content^36,37^). While in standard CT images WAT, BAT, and muscles can be easily differentiated based on their relative water/fat content, thus radiodensity (WAT is less radiodense than BAT, which is less radiodense than muscle), blood and muscles cannot. This necessitates the use of intravenous contrast agents, with atoms that have a relative high Z-number and x-ray attenuation properties, for CT angiography. These agents significantly increase the blood radiodensity, making blood vessels stand up from nearby tissues.

Here we perform CT angiography with a relatively new gold-based blood pool contrast (Mvivo from MediLumine, Quebec, Canada) that has lower vasculature permeability and much longer circulation time than Iohexol, an iodine-based CT contrast agent used widely in clinical practice. The lower vasculature permeability of Mvivo guarantees that the agent stays in the vasculature, without changing the radiodensity of nearby tissue, while its longer circulation time enables observation of dynamic vasculature changes that occur during adrenergic stimulation of NST in BAT.

## Methods

### Animals

Six normal healthy mice (C57/BL6, male, 7–8 weeks of age), house at normal room temperature since birth, were used in this study. All animal experiments were performed according to the protocol approved by the Institutional Animal Care and Use Committee (IACUC) of the University of North Carolina at Chapel Hill. All methods were carried out in accordance with relevant guidelines and regulations and the authors complied with the ARRIVE guidelines.

### CTA imaging protocol

In vivo CTA studies were performed on six C57 male mice using a microCT scanner, Quantum GX2 system (Perkin Elmer, Waltham, Massachusetts). The CT imaging parameters were 90 kVp of peak voltage, 88 µA of current, standard resolution with 120 µm nominal resolution, 41 mm FOV for one bed acquisition. For imaging, the mice were first sedated with an intraperitoneal injection of 70 mg/kg pentobarbital. After anesthesia induction a first CT scan was performed. At the end of the pre-contrast scan, the mice were intravenously injected with 100 µL of 200 mg/ml solution of the Mvivo blood pool contrast agent (MediLumine, Quebec, Canada). The Mvivo contrast agent is a relatively new gold-nanoparticle based blood pool contrast agent with core particle size of 15 nm. Compared to the other gold nanoparticle-based contrast agent, Aurovist, Mvivo showed much longer circulation time (only about 5% concentration reduction after 4 h of circulation time) with similar enhancement power (data not shown). Five minutes after the injection of Mvivo, a second CT scan was performed. The mice were then either injected subcutaneously with a 0.1 ml dose of norepinephrine (n = 3, 1 mg/kg, subcutaneous) or a 0.1 ml dose of CL-316,243 (n = 3, 1 mg/kg, subcutaneous). A third CT scan was then performed ten minutes post injection. Finally, the mice were euthanized with an overdose of pentobarbital (200 mg/kg), and a high-resolution postmortem scan was performed with 40 µm isotropic nominal resolution to be achieved in the region of iBAT.

### Image analysis

**Figure 1:**
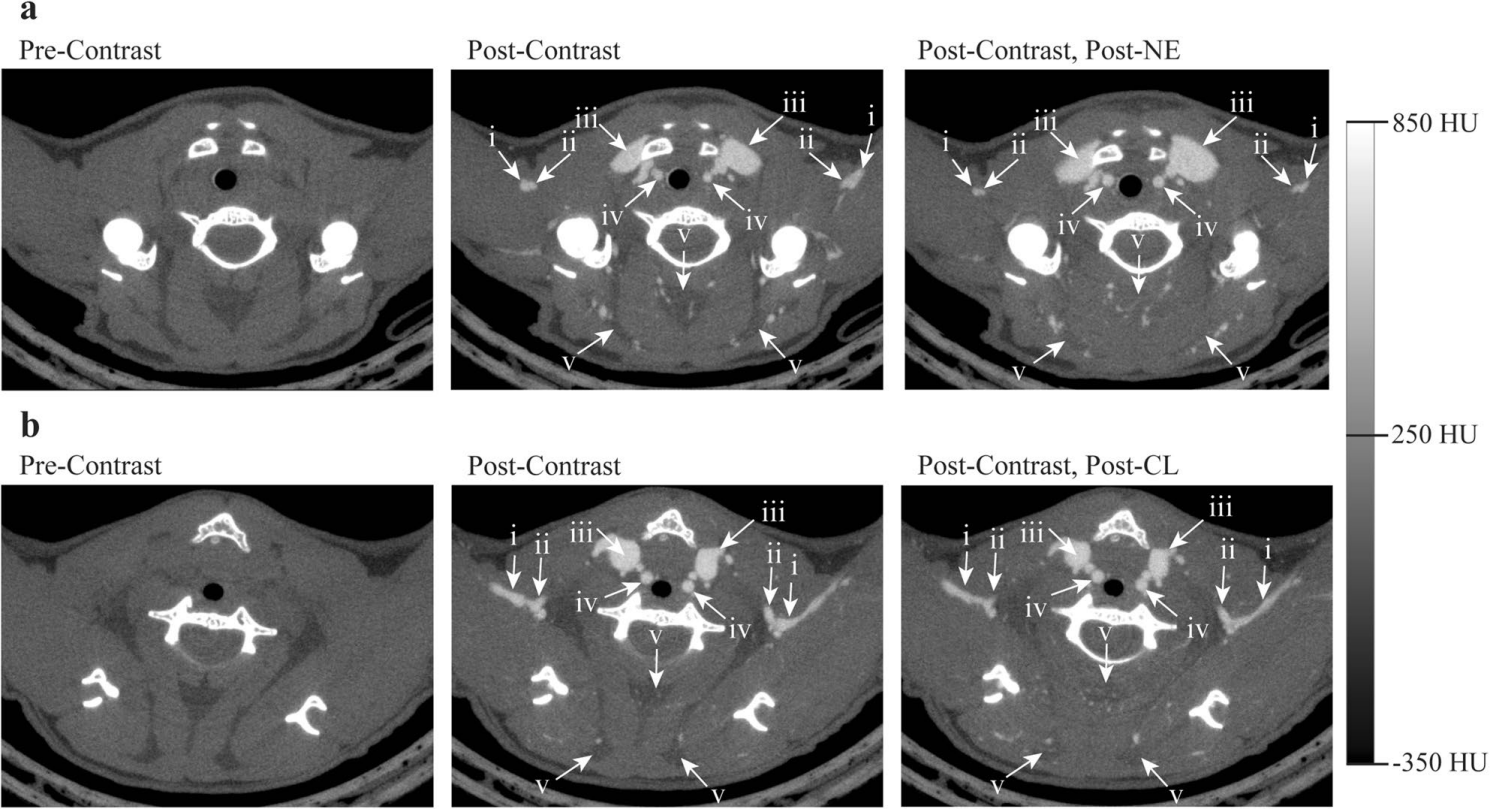
Representative CT axial images of the cervical region showing changes in vascular tone following administration of NE (**a**) and CL (**b**). A blood radiodensity enhancement of more than 500 HU was observed in all animals after administration of the CT contrast. After norepinephrine administration (**a**), the left (L) and right (R) axillary veins (i) constricted from a diameter of 0.39 mm to 0.23 mm and from 0.36 to 0.18 mm, respectively. The axillary arteries (ii) constricted from 0.22 to 0.15 mm (L) and from 0.18 to 0.13 mm (R). The jugular veins (iii) dilated from 0.93 to 1.36 mm (L) and 1.18 to 1.57 (R). The carotids arteries (iv) dilated from from 0.31 to 0.49 mm (L) and 0.35 mm to 0.45 mm (R). An increase in radiodensity due to vasodilation of unresolvable vessels in the cervical BAT can also be observed (v). Similar vascular changes are not observed after CL administration (**b**), but there is a visible radiodensity increase in the cervical BAT due to vasodilation of unresolvable vessels. All images are displayed using the same Hounsfield scale, ranging from – 350 HU to + 850 HU.

CTA images were analyzed using the open-source software package 3D slicer^38^. Gross vasculature structures were identified using anatomic landmarks and segmented from the full body scans and subvolume reconstructions using region growing (Fig. 1). Changes in tissue radiodensity between animals treated with NE and CL were compared by using a heteroscedastic t-tests JMP (Version 16.1.0, SAS Institute Inc., Cary, NC) software (Table 1). Major vessels with a diameter of approximately at least 1 mm were included in the full-body region segments, except for intestinal vessels that were intentionally excluded for visualization of the underlying vasculature. Vessels greater than approximately 0.2 mm were included in the 40 µm resolution subvolume reconstruction region growing segments.

**Table 1:** Mean ± SD radiodensity change measured in iBAT after the injection of the IV contrast and after treatment for the two groups.

	Radiodensity change of iBAT (mean HU ± SD)		p value
	NE	CL	
Post contrast-pre contrast	62.70 ± 15.42	71.46 ± 7.23	0.44
Post treatment-post contrast	89.57 ± 21.14	38.78 ± 13.11	0.03*

The change in radiodensity that followed the IV contrast injection was similar between the two sets of mice. However, an additional and significantly greater increase in tissue radiodensity was subsequently observed in mice following treatment with NE compared to mice treated with CL (p = 0.03).

Changes in vascular diameter in response to NE or CL were measured in several significant vascular structures in each animal (Table 2). The diameter of Sulzer’s vein was measured at its trunk just posterior to the thoracic vertebrae. The diameters of the left and right thoracodorsal veins were measured lateral to its bifurcation in iBAT. The diameters of the left and right dorsal cervical veins were measured at the location where it travels in the inferior-superior direction, perpendicular to the axial plane. The left and right carotid arteries and jugular veins were measured just inferior to the clavicles. The left and right axillary veins were measured lateral to the branching of the mammary vein from the subclavian. The left and right tail veins were measured at the level of the ischial tuberosity. One measurement was taken from Sulzer’s vein in each animal treated with NE (n = 3) or CL (n = 3), and one measurement was taken from each of the left and right vessels for the remaining vasculature structures (n = 6 each for NE and CL). Heteroscedastic t-tests were performed using JMP (Version 16.1.0, SAS Institute Inc., Cary, NC) software to test for differences in changes in vessel diameters between NE and CL treatment groups.

**Table 2:** Mean ± SD changes in vascular diameter in response to NE or CL were measured in several significant vascular structures.

	% change in vessel diameter following NE (mean ± SD)	% change in vessel diameter following CL (mean ± SD)	p value
Sulzer’s vein	117.3 ± 34.6	75.2 ± 31.3	0.1939
thoracodorsal vein	94.5 ± 35.7	70.3 ± 23.0	0.1967
Dorsal cervical vein	84.2 ± 16.9	77.9 ± 27.5	0.6487
Carotid artery	65.2 ± 37.9	− 0.1 ± 7.1	0.0077*
Jugular vein	37.1 ± 11.8	1.7 ± 8.0	0.0002*
Axillary vein	− 35.1 ± 9.6	0.8 ± 9.2	< 0.0001*
Tail vein	− 47.4 ± 11.9	− 14.8 ± 10.8	0.0006*

One measurement was taken from Sulzer’s vein in each animal treated with NE (n = 3) or CL (n = 3), and one measurement was taken from each of the left and right vessels for the remaining vasculature structures (n = 6 each for NE and CL).

Significantly greater vasodilation occurred in the carotid arteries and jugular veins following NE compared to CL. Significantly greater vasoconstriction occurred in the axillary veins and tail veins following NE compared to CL.

Relative changes in iBAT blood volume following the injection of NE and CL were quantified by segmenting all visible vasculature of iBAT by applying a threshold with a minimum of 180 HU. The 180 HU threshold was chosen because it allowed the inclusion of most of the resolvable vessels while excluding the surrounding tissue. JMP software was then used to perform a heteroscedastic t-test to assess differences due to the two treatments using JMP software.

## Results and discussion

### In vivo vascular changes

**Figure 2:**
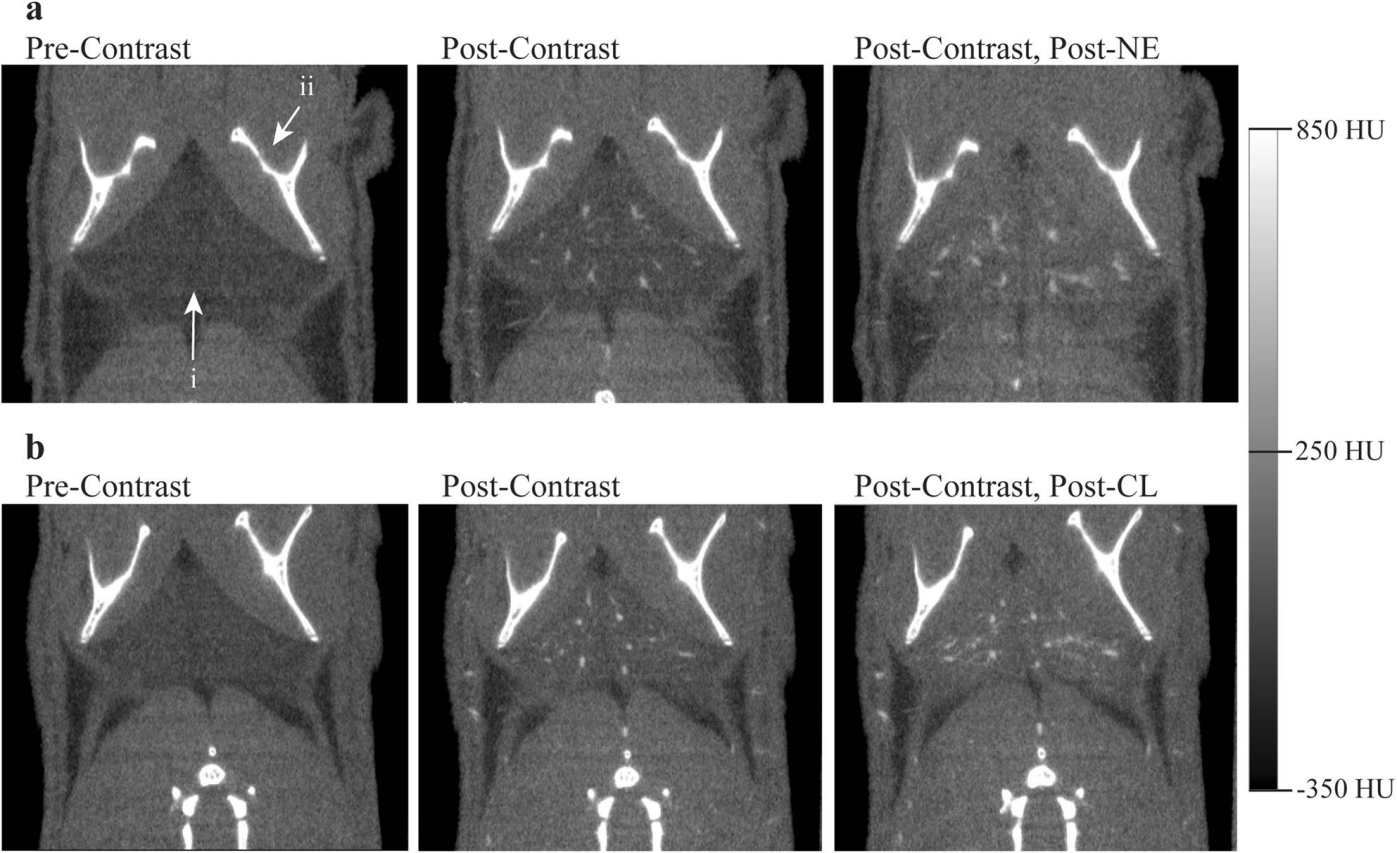
Representative coronal images of the iBAT region (i) inferior to the scapula (ii) showing vasodilation of larger blood vessels as well as radiodensity enhancement due to dilation of unresolved small vessels following treatment with both NE (**a**) and CL (**b**). Radiodensity enhancement, and consequently relative blood volume increase, was visibly greater following treatment with NE compared to CL. All images are displayed using the same Hounsfield scale, ranging from – 350 HU to + 850 HU.

**Figure 3:**
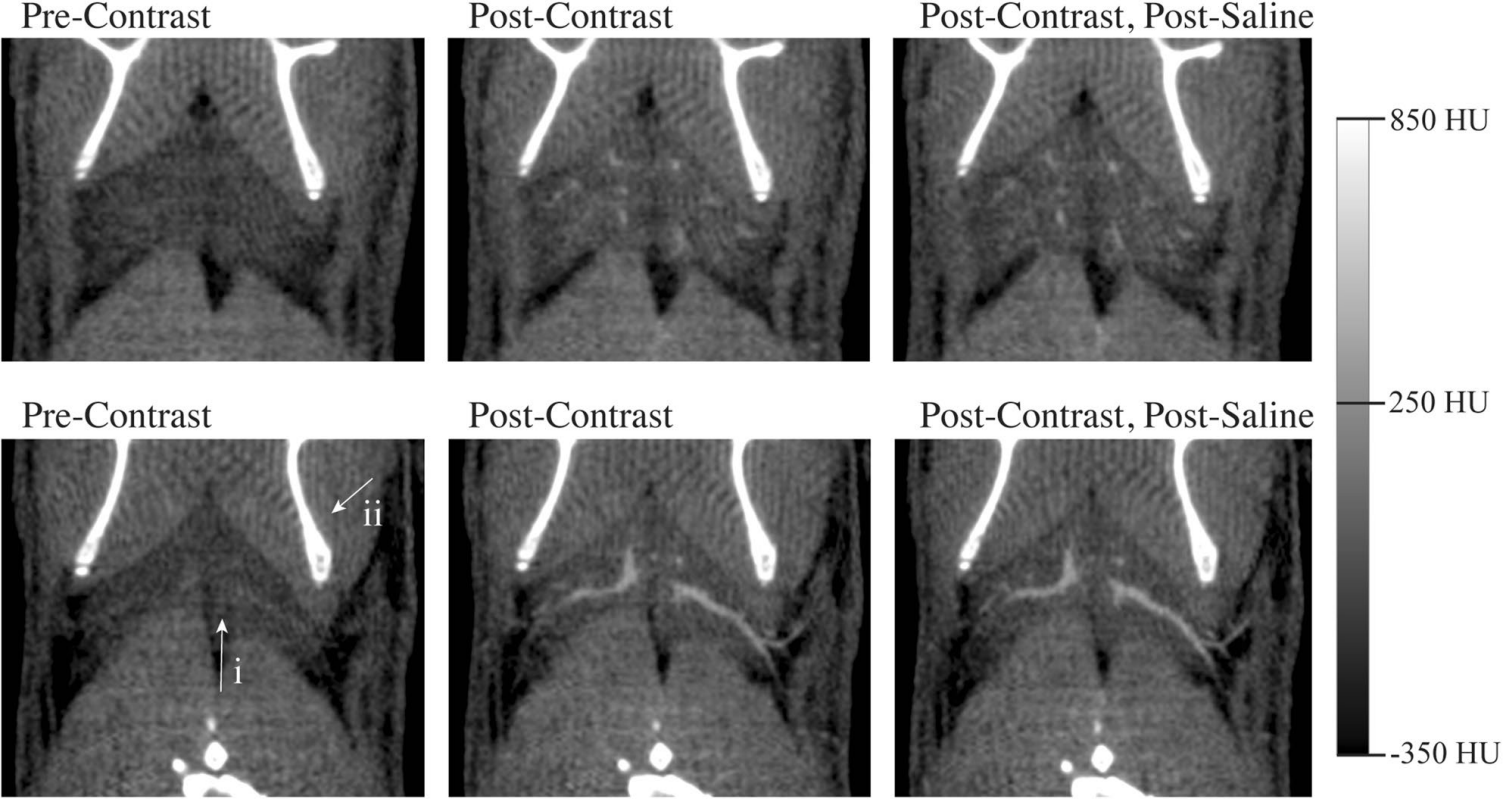
Representative coronal images of the iBAT region (i) inferior to the scapula (ii) showing no vasodilation of larger blood vessels or radiodensity enhancement following treatment with saline. No radiodensity enhancement, or changes in relative blood volume are observed before and after saline injection. Images are displayed using the same Hounsfield scale, ranging from – 350 HU to + 850 HU.

Through the examination of CTA, changes in vascular tone, or lack thereof, were identified throughout the bodies of mice treated with both NE and CL. Figure 1 shows representative CT axial images acquired before intravenous contrast injection, right after intravenous contrast injection, and after NE and CL injection. In this view of the cervical region, the images clearly show vasodilation of the jugular veins and carotids arteries, vasoconstriction of the axillary arteries and veins, and an overall radiodensity enhancement—an increase in radiodensity observed as increased brightness on CT—in the cervical BAT following NE injection (Fig. 1a). Because the Mvivo contrast does not extravasate, any increase in tissue radiodensity can be attributed to both an increase in tissue blood volume, i.e. to an increase in vasodilation of both large visible vessels and small unresolvable vessels, as well as to a decrease in tissue fat content. However, the latter is expected to be quite small and on the order of 10–15 HU for mice housed at normal room temperature and with an already relatively hydrated iBAT^36^. The large radiodensity enhancement observed in the cervical BAT therefore can be attributed mainly to the vasodilation of small vessels that are not resolved with the current image resolution. Interestingly, although some radiodensity enhancement in the cervical BAT is visible following the injection of CL, the visible vasculature in this region appears to be largely unchanged (Fig. 1b). Focusing on the interscapular BAT depot in Fig. 2, visible vasodilation as well as radiodensity enhancement were evident following the injection of both NE and CL. Just as in the cervical depot, this intense radiodensity enhancement, which is not observed in nearby WAT, was mainly a result of the vasodilation of small vessels in BAT, with some small contribution from the decrease in tissue fat content. The injection of equal concentrations of contrast should have the same effect on BAT radiodensity on each of the treatment groups if the baseline blood volume in the BAT is the same for both groups, and this was confirmed upon measuring the mean increases in tissue radiodensity following IV contrast administration in each group (Table 1, first row). However, despite there being an additional visible increase in iBAT radiodensity following both treatments with NE and CL (Fig. 2), the additional increase in tissue radiodensity following NE treatment was significantly higher than the increase seen after CL treatment (Table 1, second row). Control experiments were also performed by injecting, in different mice, the same volume of saline subcutaneously. In this case no changes in vasculature diameters or tissue radiodensity were observed (Fig. 3).

**Figure 4:**
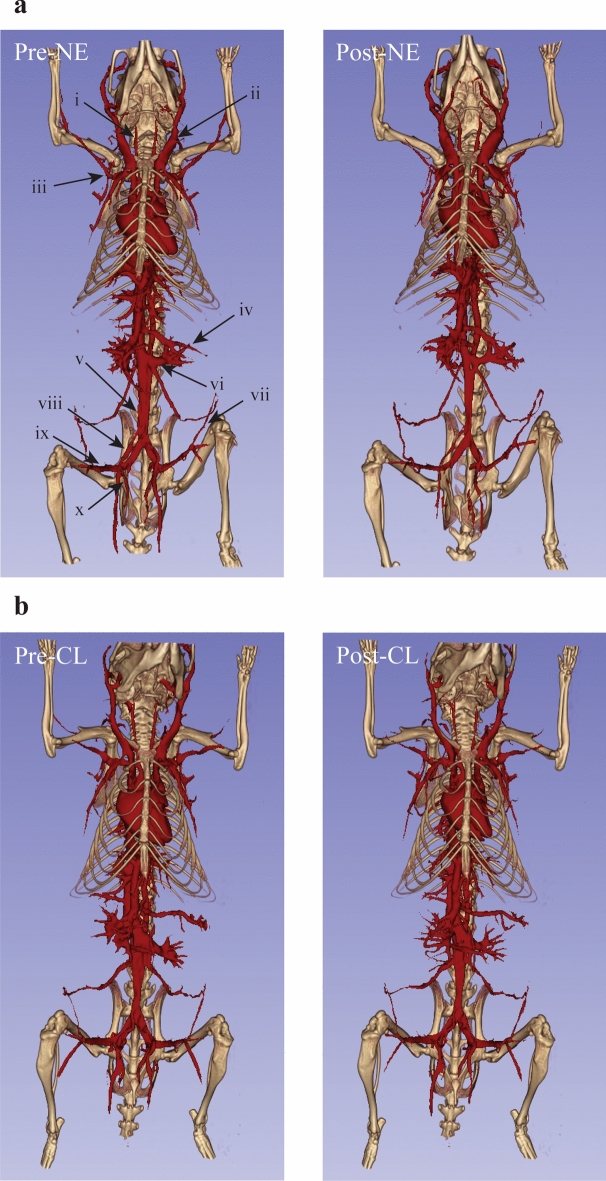
Full body vascular segmentations demonstrate the reactivity of major arteries and veins to NE and CL. Major vascular structures include the carotid arteries (i), jugular veins (ii), axillary arteries and veins (iii), splenic vein (iv), descending aorta and inferior vena cava (v), renal veins (vi), genital veins (vii), and the common (viii), external (ix), and internal (x) iliac arteries and veins.

Full body segmentation of the vasculature revealed distinct differences in the systemic vascular responses between mice treated with NE and CL (Fig. 4). Following injection with NE, mice experienced significant peripheral vasoconstriction (Fig. 4a). In the lower extremities, vasoconstriction occurred in the common, internal and external iliac arteries and veins. Similar vasoconstriction was not observed in mice treated with CL (Fig. 4b). Vasoconstriction was noted in the tail veins, an important thermoregulatory organ, of both mice treated with NE and CL. However, vasoconstriction was significantly greater in mice treated with NE compared to CL Table 2). In the abdomen, vasoconstriction following NE was observed in the inferior vena cava and descending aorta as well as in the splenic, gastric, mesenteries, and renal vessels. No changes were observed in these vessels following treatment with CL. Vascular changes in the genital arteries and veins coursing through the inguinal fat depot were not observed following treatment with either NE or CL. Interestingly this depot is one of the well-known WAT depots that can undergo browning following chronic cold or CL exposure. More cranially, vasodilation of the internal and external jugular veins in addition to the carotid artery were observed following treatment with NE, but no changes were observed after treatment with CL.

**Figure 5:**
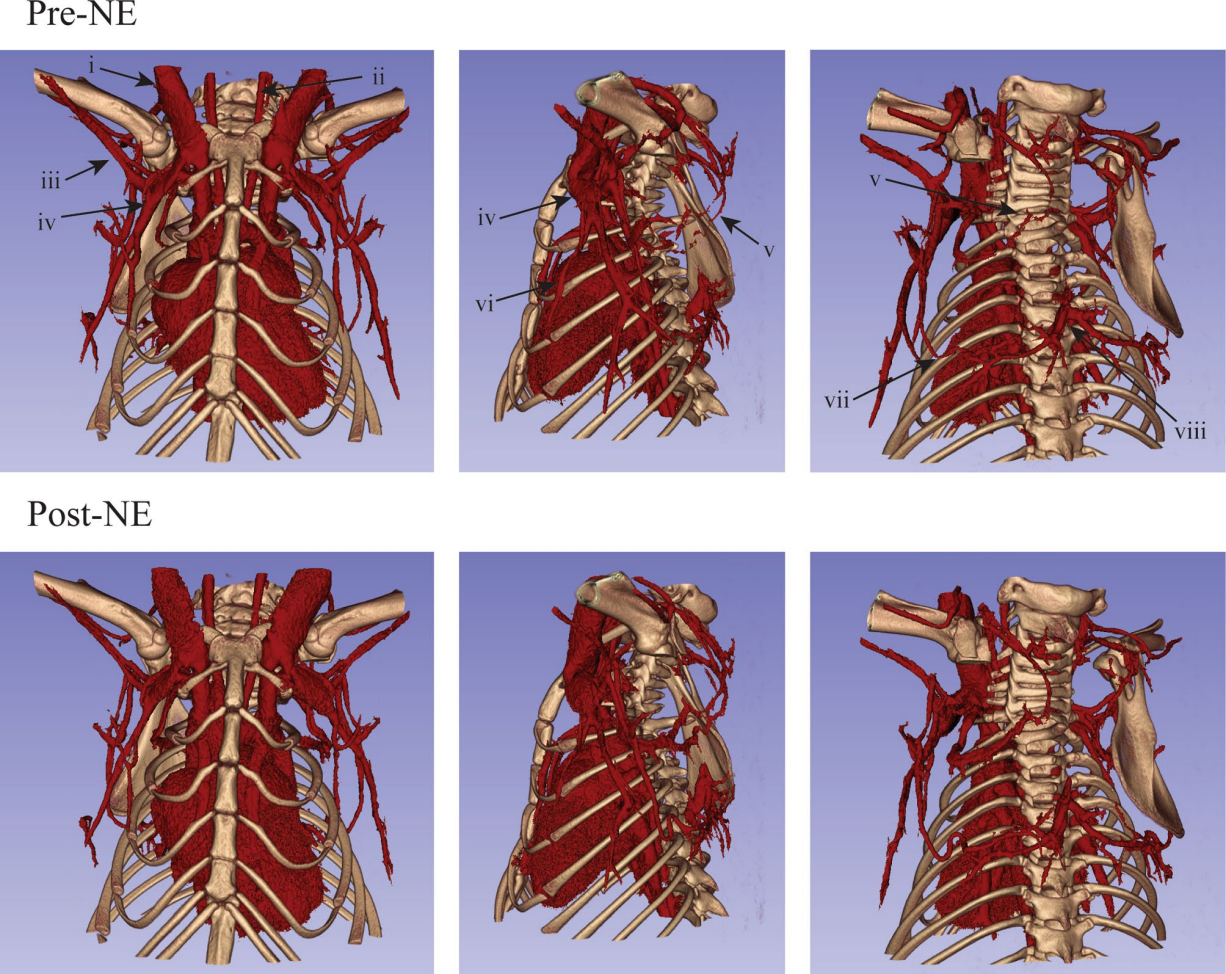
40 µm resolution vascular segmentations of the upper body demonstrate vascular reactivity to NE in the region of cervical and interscapular brown adipose tissue. Notable vascular structures include the jugular veins (i), carotid arteries (ii), axillary arteries and veins (iii), lateral thoracic veins (iv), dorsal cervical arteries and veins (v), mammary veins (vi), thoracodorsal arteries and veins (vii), and Sulzer’s vein (viii).

**Figure 6:**
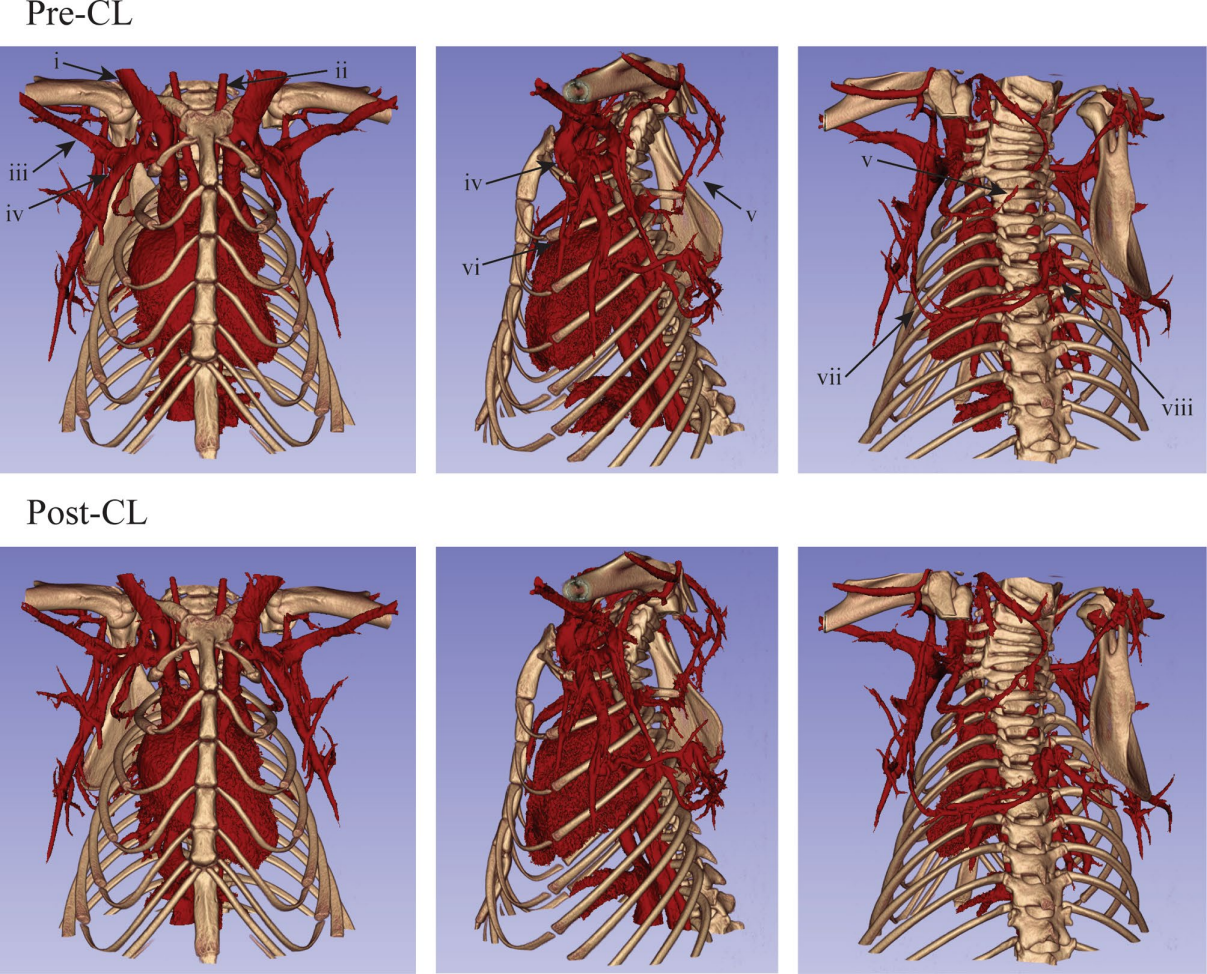
40 µm resolution vascular segmentations of the upper body demonstrate vascular reactivity to CL in the region of cervical and interscapular brown adipose tissue. Notable vascular structures include the jugular veins (i), carotid arteries (ii), axillary arteries and veins (iii), lateral thoracic veins (iv), dorsal cervical arteries and veins (v), mammary veins (vi), thoracodorsal arteries and veins (vii), and Sulzer’s vein (viii).

The 40 µm reconstructions of the thoracic and cervical areas of the mice yielded the best visualization of vascular reactivity in that region (Figs. 5 and 6). The anterior view provides a better view of the NE-induced vasodilation of the jugular veins and carotid arteries (Fig. 5) and lack of reactivity to CL (Fig. 6, Table 2). Additionally, vasodilation of the lateral thoracic vein just lateral to the first rib and vasoconstriction of the axillary arteries and veins were observed following treatment with NE but not CL (Table 2). The lateral view revealed vasodilation of the thoracodorsal artery and vein in mice treated with both NE and CL (Table 2). The view also shows vasoconstriction of the mammary artery and vein following NE treatment but not after CL. Both the lateral and posterior views showed significant vasodilation of the dorsal cervical arteries and veins feeding the cervical brown adipose tissue after treatment with NE and CL (Table 2). Prior to stimulation of BAT, the dorsal cervical vessels are nearly invisible where the superior and inferior branches meet. This connection becomes evident following vasodilation after both NE or CL injection. Additionally, vasodilation of Sulzer’s vein and the thoracodorsal arteries and veins within the iBAT is visible in the posterior view after treatment with both NE and CL.

**Figure 7:**
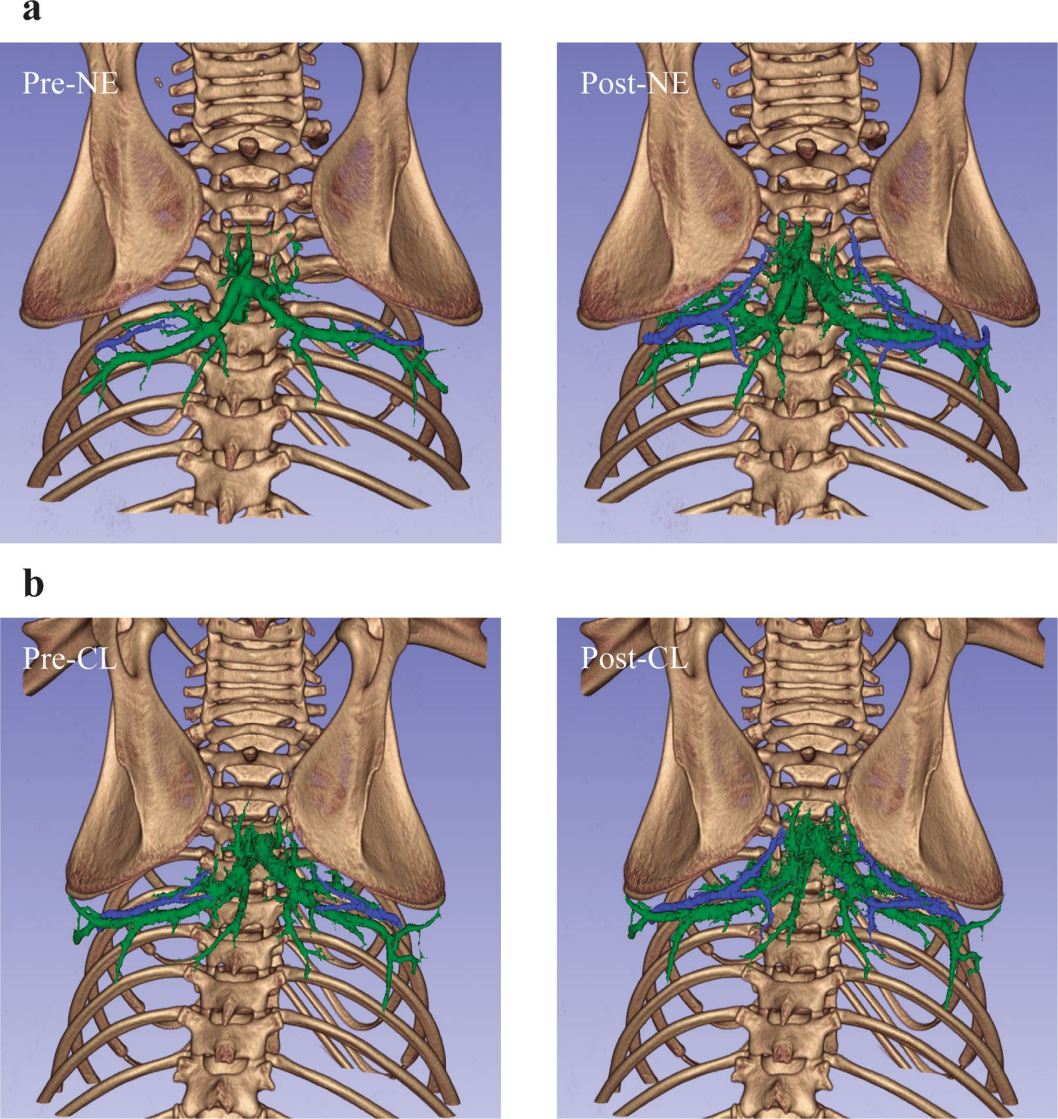
40 µm resolution vascular segmentations in interscapular brown adipose tissue show vasodilation of the thoracodorsal arteries and veins (blue) as well as the Sulzer’s vein (green). The left and right thoracodorsal veins dilated from 0.20 to 0.29 mm and from 0.19 to 0.27 mm, respectively, following the injection of NE (**a**). Following CL injection, the left and right thoracodorsal veins dilated from 0.17 to 0.21 mm and from 0.17 to 0.22 mm, respectively (**b**).

A more detailed segmentation of the vasculature within the iBAT itself demonstrated the size of vessels contributing to increased perfusion in the region following NE and CL (Fig. 7). While the major branches of Sulzer’s vein do dilate to some extent, it is primarily its significant number of smaller branches and its trunk that dilate following treatment with NE or CL. The thoracodorsal artery and vein react differently, whereby the vessels are normally extremely constricted near its point of bifurcation in the animal’s resting state, and stimulation with NE or CL cause dilation of the major branches to allow the bilateral blood supply and drainage from the vessels to extend deeper into the fat.

### Relative blood volume increases

Although vasodilation of Sulzer’s vein and the thoracodorsal arteries and veins in iBAT occurred both following treatment with NE and CL, the magnitude of the resulting change in blood volume in the tissue differed between the two. The volume of large vessels increased significantly more in mice treated with NE compared to mice treated with CL (Table 3). Visible differences in iBAT radiodensity enhancement were also observed between animals treated with NE and CL (Fig. 2, Table 1), indicative of greater vasodilation of small, unresolvable vessels in animals treated with NE compared to those treated with CL (Fig. 2). Local regulation of blood flow tends to occur most significantly at the level of arterioles feeding capillaries^39,40^, so it is expected that widespread dilation of unresolvable vessels would result from BAT activation. This effect is confirmed by previous post-mortem studies that were able to resolve the microvasculature of BAT only after stimulation of NST by NE^17^, and is generally also observed in MRI scans as a decrease in tissue transverse relaxation^41,42^.

**Table 3:** Blood volumes before and after treatment with NE or CL and associated increases.

	Mean ± SD blood volume within iBAT (mm^3^)		p value
	NE	CL	
Pre-treatment	3.24 ± 0.74	4.42 ± 1.08	–
Post-treatment	8.72 ± 1.85	6.66 ± 2.02	–
Increase	5.47 ± 1.34	2.24 ± 0.98	0.03*

Blood volumes were measured by applying a threshold of 180 HU to manually segmented iBAT and counting the voxels above the threshold. A significantly greater increase in blood volume was seen in mice treated with NE compared to those treated with CL (p = 0.03).

**Figure 8:**
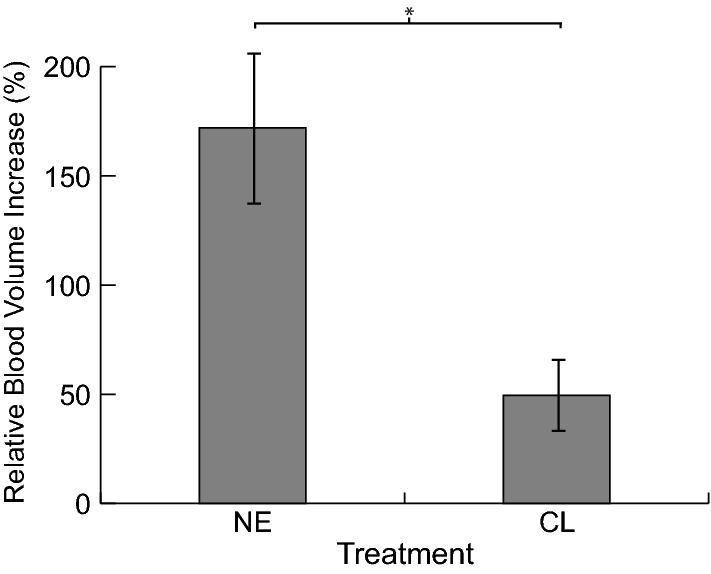
Mean relative blood volume increase measured in iBAT after NE and CL treatment. Blood volume increase was measured to be 172% ± 49% (mean ± SD) in animals treated with NE and 49% ± 13% in animals treated with CL (p = 0.03). Blood volume increase was significantly higher in mice treated with NE compared to mice treated with CL. Error bars represent standard deviation.

A pixel voxel count of the segmented vasculature demonstrated significantly higher (p = 0.03) relative increase in iBAT blood volume after treatment with NE (Mean ± SD of 172% ± 49%) than after treatment with CL (49% ± 13%) (Fig. 8).

The relative tissue blood volume changes observed in these studies clearly demonstrate that a difference exists in how NE and CL impact vascular changes in iBAT. This difference may be explained by the systemic effect elicited by these molecules. NE and CL both activate BAT by binding with β_3_ adrenergic receptors^8,24,25,27,28^, which may cause local vasodilation due to increased production of NO by BAT^29,30,32^. However, CL’s high specificity to β_3_ adrenergic receptors limits its impact on vasculature in other areas of the body^24^. NE, on the other hand, binds much less selectively to all α and β adrenergic receptors^22,23^. The result is an extensive peripheral vasoconstriction that we observed very clearly with high-resolution in vivo CTA, and that likely led to greater blood flow diversion to iBAT. It was recently shown that acute CL exposure did not result in increased oxygen consumption in the BAT of rats regardless of dose when compared to control, while acute treatment with 1 µM or 10 µM NE did result in increased oxygen consumption^43^. These data seem to corroborate our findings and suggest a correlation between increase in tissue blood volume and oxygen consumption.

An important limitation of this study is that intravenous administration of NE and CL induces systemic effects and may not fully recapitulate the physiological changes that occur under natural stimulation of NST by cold. To overcome this limitation, one may think of performing similar experiments while exposing animals to cold temperatures. However, it is important to consider that, for our high-resolution studies, animals must be anesthetized to a surgical plane to limit motion. When the animal is under a surgical plane of anesthesia its ability to regulate its body temperature through NST is completely lost. In other words, even if BAT were to be activated by cold before anesthesia induction, it would no longer be active once the animal is brought down to a surgical plane of anesthesia. This is true even with pentobarbital anesthesia, one of the few anesthetics that is known not to interfere with BAT metabolism^44^. One could then think of performing similar experiments in awake mice, using paralytic agents to limit motion. However, even for these studies (which necessitate special regulatory exemptions), anesthesia or anxiolytics agents would be needed to limit stress response. Alternatively, if anesthesia and/or anxiolytic agents were not used, it would be unclear how to decouple the effect of cold from acute stress^45^.

## Conclusion

We have demonstrated the utility of in vivo CT angiography with Mvivo Au blood pool contrast for studying vascular reactivity in small animals during stimulation of NST by catecholamines. By examining CT images and 3D vascular segments created from them, we found that while both NE and CL lead to increased blood volume in BAT through local vasodilation, NE causes a greater increase, likely through the concurrent peripheral vasoconstriction. Given the different adrenergic receptors targeted by these two molecules, our findings are consistent with the expected physiological response to these drugs. This technique has a wide range of potential applications such as studying mice with altered thermoregulatory profiles due to defects in the cardiovascular system. Additionally, the use of CT with alternative contrast agents like Iodine could facilitate the study of BAT vasculature reactivity in humans.

## Data Availability

The dataset supporting the conclusions of this article is available in the Carolina Digital Repository. https://cdr.lib.unc.edu/collections/7h14b072w?locale=en.
